# Dynamic Contrast-Enhanced Magnetic Resonance Imaging with Gd-EOB-DTPA for the Evaluation of Liver Fibrosis Induced by Carbon Tetrachloride in Rats

**DOI:** 10.1371/journal.pone.0129621

**Published:** 2015-06-15

**Authors:** Wei Zhang, Xiang Kong, Zhen J. Wang, Song Luo, Wei Huang, Long Jiang Zhang

**Affiliations:** 1 Department of Medical Imaging, Jinling Hospital, Medical School of Nanjing University, Nanjing, Jiangsu, 210002, China; 2 Department of Radiology and Biomedical Imaging, University of California San Francisco, San Francisco, CA, United States of America; Brandeis University, UNITED STATES

## Abstract

**Purpose:**

To investigate the utility of dynamic contrast-enhanced MRI (DCE-MRI) with Gadolinium ethoxybenzyl diethylenetriamine pentaacetic acid (Gd-EOB-DTPA) for detecting liver fibrosis induced by carbon tetrachloride (CCl_4_) in rats.

**Methods:**

This study was approved by the institutional animal care and use committee. Liver fibrosis in rats was induced by intraperitoneal injection of 1 mL/kg 50% CCl_4_ twice a week for 4-13 weeks. Control rats were injected with saline. Liver fibrosis was graded using the Metaviar score: no fibrosis (F0), mild fibrosis (F1-F2) and advanced fibrosis (F3-F4). DCE-MRI with Gd-EOB-DTPA was performed for all rats. K^trans^, K_ep_, V_e_ and iAUC of the liver parenchyma were measured. Relative enhancement (RE) value of the liver was calculated on T_1_-weighted images at 15, 20 and 25 min after Gd-EOB-DTPA administration.

**Results:**

Thirty-five rats were included: no fibrosis (n=13), mild fibrosis (n=11) and advanced fibrosis (n=11). K^trans^ and iAUC values were highest in advanced fibrosis group and lowest in no fibrosis group (*P＜*0.05). The area under the receiver operating characteristic curve (AUROC) for fibrosis (stages F1 and greater) were 0.773 and 0.882 for K^trans^ and iAUC, respectively. AUROC for advanced fibrosis were 0.835 and 0.867 for K^trans^ and iAUC, respectively. K_ep_ and RE values were not able to differentiate fibrosis stages (all *P*＞0.05).

**Conclusion:**

K^trans^ and iAUC obtained from DCE-MRI with Gd-EOB-DTPA are useful for the detection and staging of rat liver fibrosis induced by CCl_4_.

## Introduction

Liver fibrosis is a common feature of almost all causes of chronic liver disease, and eventually leads to cirrhosis [1, 2]. Among digestive diseases, cirrhosis is the most common nonneoplastic cause of mortality and it is also a risk factor for hepatocellular carcinoma [3]. In recent years, increasing research has suggested that liver fibrosis is reversible, especially in the early stage [2, 4, 5]. Therefore, early and accurate diagnosis and staging of liver fibrosis is critical in allowing early treatment and in the prevention of progression to cirrhosis.

Liver biopsy is the reference standard for the diagnosis and staging of liver fibrosis. However, it is not suitable for screening, long-term monitoring and assessing therapeutic response due to its invasiveness, costs, and sampling variability [6]. These drawbacks have led to the development of an increasing number of imaging-based methods for noninvasive assessment of liver fibrosis, including ultrasound elastography [7], computed tomography with macromolecular contrast material [8], as well as magnetic resonance imaging (MRI) based techniques [6,9–11]. Among these techniques, MRI is a promising tool with several advantages, including being non-ionizing, non-invasive, offering high spatial resolution and multiparameter imaging capability.

The most important pathological changes of liver fibrosis are sinusoidal capillarization and deposition of collagen, proteoglycans, and other macromolecules in the extracellular matrix [1, 2, 4]. These changes impede the rapid exchange of solutes between sinusoid and hepatocytes and expand the extravascular extracellular space (EES) [8, 9]. Dynamic contrast-enhanced MRI (DCE-MRI) is a MR imaging technique which can assess the microcirculation perfusion status of tissues [12]. Using this technique, several quantitative parameters including transfer constant (K^trans^), rate constant (K_ep_), extravascular extracellular space volume fraction (V_e_), and semi-quantitative parameters such as the initial area under the gadolinium concentration-time curve (iAUC), can be measured [13, 14]. DCE-MRI has been used to diagnose, predict prognosis, and monitor treatment response in a variety of tumors [15–19]. Gadolinium ethoxybenzyl diethylenetriamine pentaacetic acid (Gd-EOB-DTPA) is a hepatocyte-specific contrast agent for MRI. After bolus injection into vein, about 50% of Gd-EOB-DTPA is taken up by functioning hepatocytes and then excreted through the biliary system [20]. The other 50% will return into blood vessels from EES and is eliminated by kidney [20]. Therefore, Gd-EOB-DTPA combines the features of an extracellular contrast agent and a hepatocyte-specific agent and can provide both morphologic information of liver and functional information of hepatocytes at the same time [9, 20]. Hepatocyte functions are impaired in liver fibrosis due to increased necroinflammatory changes [21]. Therefore, we undertook this study to test the hypothesis that DCE-MRI with Gd-EOB-DTPA can be used to assess the presence and severity of liver fibrosis, using a rat model of liver fibrosis induced by carbon tetrachloride.

## Materials and Methods

### Animal Model

**Fig 1:**
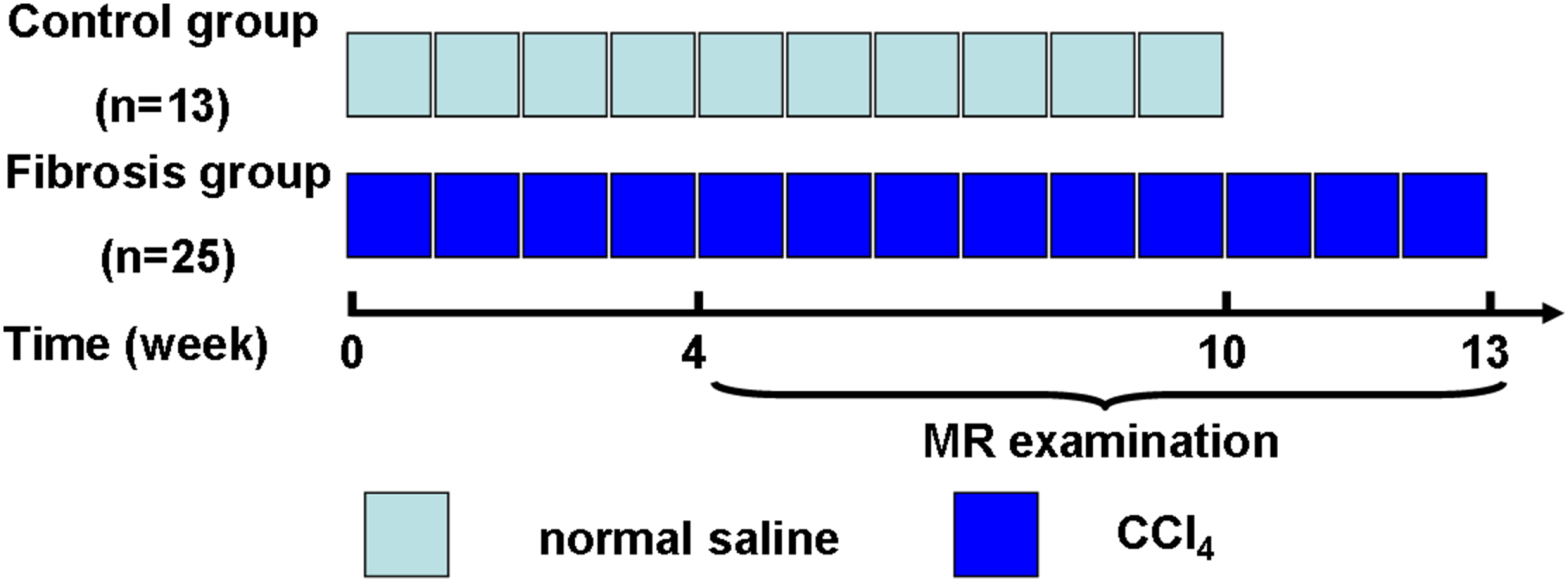
Scheme of this experimental study. Rats were treated by intraperitoneal injection of normal saline [control group (n = 13)] or CCl_4_ [fibrosis group (n = 25)] (50% CCl_4_/olive oil; 1 mL/kg body weight twice a week) for 4 to 13 weeks to produce different degrees of liver fibrosis. Starting from the fourth week, two or three rats in each group were examined in a 3 Tesla MR scanner. CCI_4_ = carbon tetrachloride.

This study was approved by the institutional animal care and use committee of Jinling Hospital, Medical School of Nanjing University, Nanjing, China and was performed in accordance with institutional guidelines. Thirty-eight adult male Wistar rats (Liaoning Changsheng Biotechnology Company, Benxi, China) initially weighing 180–200g were housed in conventional cages with free access to standard laboratory food and water at 20–22°C with a 12-h light-dark cycle. Rats were treated by intraperitoneal injection of normal saline [control group (n = 13)] or CCl_4_ [fibrosis group (n = 25)] (50% CCl_4_/olive oil; 1 mL/kg body weight twice a week; Monday and Thursday) for 4 to 13 weeks to produce different degree of liver fibrosis (Fig 1).

### DCE MR Imaging

Beginning the fourth week after the start of CCl_4_ treatment, two or three rats of each group which selected randomly were examined in a 3 Tesla MR scanner (Magnetorn Trio; Siemens Medical Solutions, Erlangen, Germany) using a custom-built animal coil (Siemens, 4 channel, model No.: 10499125, Serial/lot No.:1005) at each weekend (Fig 1). Rats were fasted for 8 hours before MR scans.

All scans were performed under general anesthesia, which was initiated in an induction chamber using a mixture of 4% isoflurane (Keyuan Pharmaceutical Co., Jinan, China) and 96% oxygen, and then maintained with an animal nose mask supplying a mixture of 2% isoflurane and 98% oxygen at a flow rate of 0.8 L/min. In order to reduce respiratory movement, each rat’s abdomen was wrapped with gauze before being positioned prone in the animal coil. The precontrast transverse T_1_ weighted imaging (T_1_WI) was performed with a turbo spin echo (TSE) sequence (repetition time (TR)/echo time (TE) = 805/13 msec, flip angle (FA) = 150°, field of view (FOV) = 53×53 mm^2^, slice thickness = 2.5 mm/0.25 mm gap). The DCE-MRI data were acquired using a free-breathing three-dimensional volumetric interpolated breath-hold examination (3D-VIBE) sequences with the following parameters: TR/TE = 7.74/2.32 msec; FA = 12°; FOV = 70×50.75 mm^2^. The scanning process contains 60 continuous measurements lasting for 10 minutes and 27 seconds and each measurement has 16 axial slices with thickness 1.5 mm, slice gap 0.3 mm [14]. At the end of the third measurement, 0.1mL Gd-EOB-DTPA (Primovist, Bayer-Schering Pharma, Berlin, Germany) was injected through a previously inserted tail vein catheter (24G, BD, Suzhou, China) by hand, followed by a 1 mL saline flush [14]. Before the DCE acquisition, images for calculating T_1_ maps were acquired using the same sequence and parameters except for the flip angle (2° and 15°) [14]. Delayed phase of T_1_WI for calculating relative enhancement (RE) were obtained 15, 20 and 25 minutes after the injection of the contrast agent [22].

### Imaging Analysis

Image analysis was performed by two radiologists in consensus (L.Z., with 10 years of experience in abdominal MR imaging, and, W.Z., with 2 years of experience). Both radiologists were blinded to the histopathologic results.

Quantitative parameters including K^trans^, K_ep_, V_e_ as well as semi-quantitative parameter iAUC of the liver parenchyma were estimated from DCE-MR images using a commercial postprocessing software based on a modified Tofts model (Tissue 4D, Siemens Medical Solutions) installed at an image processing workstation (Syngo MMWP, Siemens Medical Solutions). The arterial input function (AIF) was measured at abdominal aorta and the venous input function (VIF) was measured at the main portal vein. Three circular regions of interest (ROI) were drawn by hand in the left lateral, right lateral, and medium lateral liver lobes to measure mean values. Care was taken to avoid large vessels, moving artifacts and any focal lesion.

The RE was calculated according to the following formula: RE = (PostSI − PreSI)/PreSI (23). PostSI is the liver signal intensity 15, 20 or 25 minutes after venous administration of contrast agents, respectively. PreSI is precontrast signal intensity of the liver. The ROIs were drawn as described above and the ROIs of each four phases were placed in identical anatomic positions for evaluations.

### Blood Markers

Blood samples were obtained by cardiac puncture after MR imaging. The anesthesia was maintained by inhalation of 2.0% isoflurane through a face mask during the procedure. After that, rats were euthanized with an intravenous overdose of chloral hydrate. The serum level of alanine transaminase (ALT), aspartate transaminase (AST), and alkaline phosphatase (ALP) were measured by spectrophotometry using commercially available kits (Jiancheng Institute of Biotechnology, Nanjing, China). The assay of the levels of serum hyaluronic acid (HA), laminin (LN), collagen type IV (IV-C) and procollagen type III (PC III) were done by radioimmunoassay (RIA) using commercially available kits (Beifang Biotechnology Research Institute, Beijing, China). The operations were performed according to the user’s manual.

### Histologic and Immunohistochemical Evaluation

For the assessment of the degree of liver fibrosis, livers were removed immediately after obtaining blood samples and fixed in 4.2~5.2% formaldehyde neutral buffer solution. The tissues were dehydrated, embedded in paraffin, cut in 5 μm sections and mounted on the slide. The hematoxylin-eosin (HE) stain and masson stain were used for histopathological examination. Three histologic slices excised from the left lateral, right lateral, and medium lateral liver lobes of each rat were evaluated. Histologic staging of fibrosis was done according to the METAVIR scoring system and divided into the following five stages: F0, no fibrosis; F1, early fibrosis, portal fibrosis without septa; F2, moderate fibrosis, portal fibrosis with rare septa; F3, severe fibrosis, numerous septa without cirrhosis; and F4, cirrhosis [24]. Liver slices were also used for evaluation of smooth muscle actin (SMA). Sections were incubated with monoclonal mouse anti-rat alpha-smooth muscle actin primary antibody (Boster bio-engineering Limited company, Wuhan, China), followed by anti-mouse biotinilated secondary antibody, streptavidin-peroxidase and finally the DAB chromogen. The sections were then stained with hematoxilin and mounted with Entellan.

### Statistical Analysis

Statistical analysis was performed by using software (SPSS, version 16.0, Chicago). Data were expressed as mean ± standard deviation or median (interquartile range) as appropriate. Data were first tested for normality by using a one-sample Kolmogorov-Smirnov test. One-way analysis of variance (ANOVA) with post hoc test was used to evaluate the level of significance of the difference in parameters of DCE-MRI, blood markers and RE values among fibrosis stages. Receiver operating characteristic curves (ROCs) were performed to assess which parameter showed the best accuracy for prediction of liver fibrosis. Areas under the ROCs (AUROCs) were used to estimate the probability of correctly predicting the degree of liver fibrosis. According to the AUROCs, the optimal cutoff values were determined for liver fibrosis staging. *P* values less than 0.05 indicated statistical significant.

## Results

**Fig 2:**
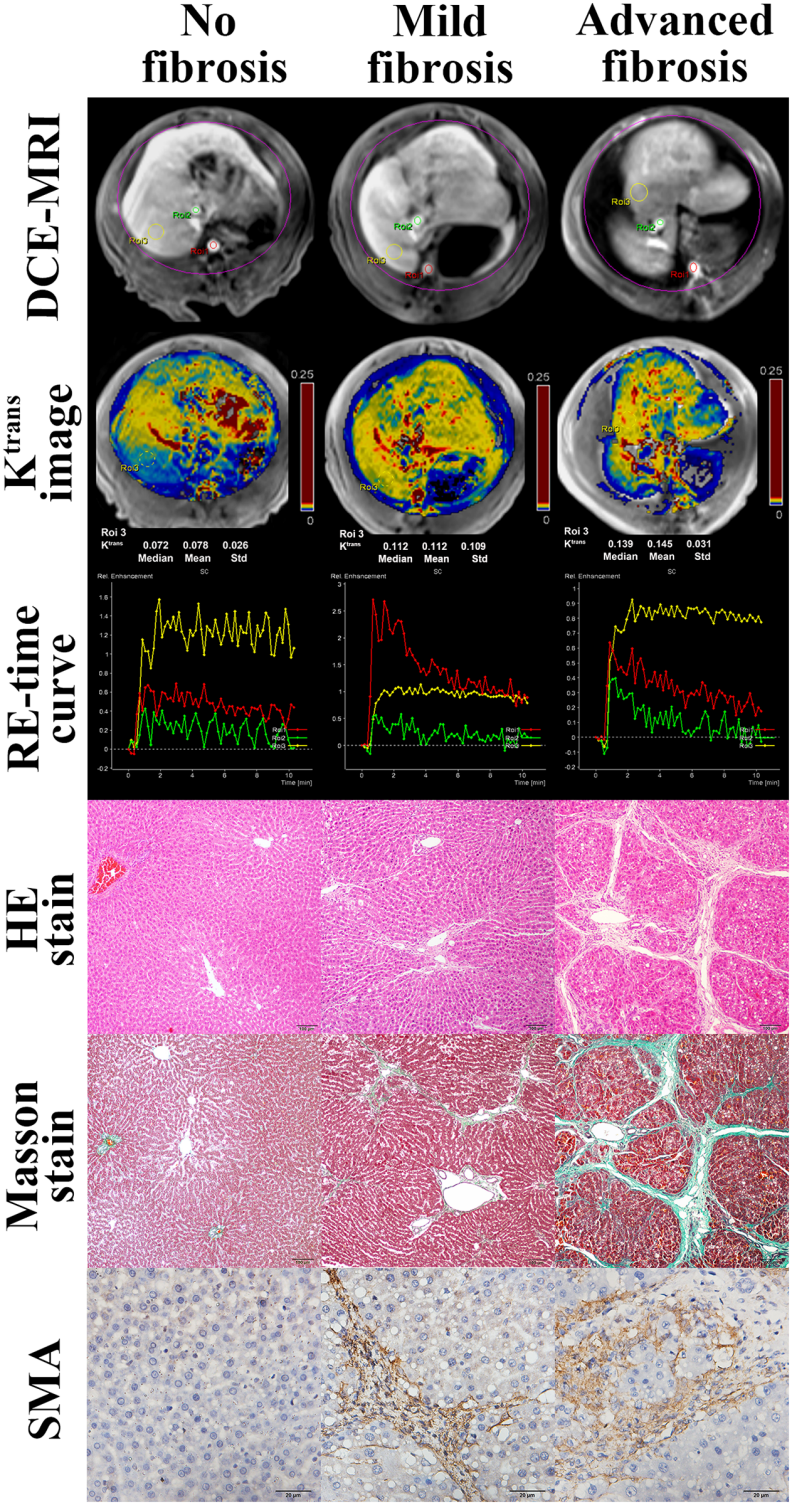
Images of DCE-MRI, K^trans^, relative enhancement-time curve, hematoxylin-eosin (HE)-stained (Original magnification, ×100), Masson trichrome-stained (Original magnification, ×100.) and SMA (Original magnification, ×400.) of normal liver, mild liver fibrosis (F2), and advanced liver fibrosis (F4) in rats. The images of DCE-MRI show the selection of ROIs of artery input function, vein input function, and liver, and the color of ROIs correspond to that of RE-time curves. DCE-MRI = dynamic contrast-enhanced MRI; HE = hematoxylin-eosin; K^trans^ = transfer constant; RE = relative enhancement; SMA = smooth muscle actin.

Three rats in the CCl_4_ treatment group died due to poor tolerance of CCl_4_. Thus, thirty-five rats were included in the study. According to histopathological results, the distribution of fibrosis stage was as follows: F0, 13 rats; F1, 1 rat; F2, 10 rats; F3, 8 rats; and F4, 3 rats. The 35 rats were then divided into three subgroups according to fibrotic stage: no fibrosis (F0, n = 13), mild fibrosis (F1 and F2, n = 11) and advanced fibrosis (F3 and F4, n = 11). The positive area of SMA stains correlated with the degree of liver fibrosis as stratified by the Metavire scores (Fig 2).

### Blood Markers

The serum level of ALT and AST increased with increasing degree of liver fibrosis. The ALT values in the no fibrosis group were lower than those in the advanced fibrosis group (*P*<0.01), while no differences were found between no fibrosis group and mild fibrosis group and between mild fibrosis group and advanced fibrosis group (both *P*>0.05). The differences in AST for each two groups (*P*<0.05) were statistically significant. The serum level of ALP decreased initially and then increased, and the differences in ALP between each two groups were not statistically significant (*P*>0.05). The serum level of HA, LN, IV-C and PC III did not show statistical differences (*P*>0.05) except the level of IV-C between no fibrosis group and mild fibrosis group (*P*<0.05) (Table 1). In the ROC analysis, ALT and AST performed well in the evaluation of liver fibrosis with an AUROC of 0.850 and 0.808 for fibrosis (stages of F1 and greater), 0.841 and 0.850 for the advanced fibrosis (stages of F3 and F4).

**Table 1:** The serum level of blood markers in different groups.

Blood markers	No fibrosis group (F0, n = 13)	Mild fibrosis group (F1-F2, n = 11)	Advanced fibrosis group (F3-F4, n = 11)
ALT (U/L)	66.08±26.53	161.09±140.91	205.27±65.03**
AST (U/L)	163.25±123.77	255.82±171.91*	511.73±350.99** ^#^
ALP (U/L)	182.50±45.63	137.55±69.67	194.46±98.63
PCIII(μg/L)	42.92±2.39	45.07±4.49	45.28±3.66
IV-C (ng/ml)	5.37±3.87	5.56±2.24*	5.39±4.18
LN (ng/ml)	75.61±10.71	70.78±9.69	78.80±14.15
HA (ng/ml)	100.93±43.44	205.05±256.07	173.64±150.47

Note.—Data are mean ± standard deviations.

*P<0.05,

** *P*<0.01 for comparison with rats in normal group.

^#^ *P*<0.05 for comparison with rats in mild group.

ALT = alanine transaminase; AST = aspartate transaminase; ALP = alkaline phosphatase; PCIII = procollagen type III; IV-C = collagen type IV; LN = laminin; HA = hyaluronic acid.

### DCE-MRI Parameters

K^trans^ and iAUC were higher with more advanced degree of liver fibrosis (Table 2 and Fig 2). K^trans^ and iAUC were significantly different between the no fibrosis and advanced fibrosis groups (both *P*<0.05) and between the mild fibrosis and advanced fibrosis groups (both *P*<0.05). With increasing degree of liver fibrosis, K_ep_ increased initially and then decreased. Although the K_ep_ for both mild and advanced fibrosis group were slightly higher than that of no fibrosis group, the difference was not statistically significant (*P*>0.05). In addition, the value of V_e_ decreased slightly initially and then increased, and the difference between no fibrosis and advanced fibrosis groups and between mild fibrosis and advanced fibrosis groups were statistically significant (both *P*<0.05).

**Table 2:** Parameters of DCE-MRI in different groups.

	No fibrosis group (F0, n = 13)	Mild fibrosis group (F1-F2, n = 11)	Advanced fibrosis group (F3-F4, n = 11)	F value	*P value*
Ktrans(min-1)	0.078±0.017	0.088±0.013	0.104±0.017** ^#^	7.991	0.002
Kep(min-1)	0.273±0.060	0.309±0.055	0.297±0.051	1.271	0.294
Ve	0.300±0.085	0.295±0.057	0.362±0.073* ^#^	2.863	0.072
iAUC	5.205±1.692	6.694±1.583	8.701±2.069** ^#^	11.409	0.000

Note.—Data are mean ± standard deviations.

*P<0.05,

**P<0.01 for comparison with rats in normal group.

^#^ *P*<0.05 for comparison with rats in mild group.

K^trans^ = transfer constant, K_ep_ = rate constant, V_e_ = extravascular extracellular volume fraction; iAUC = initial area under the gadolinium concentration-time curve.

**Fig 3:**
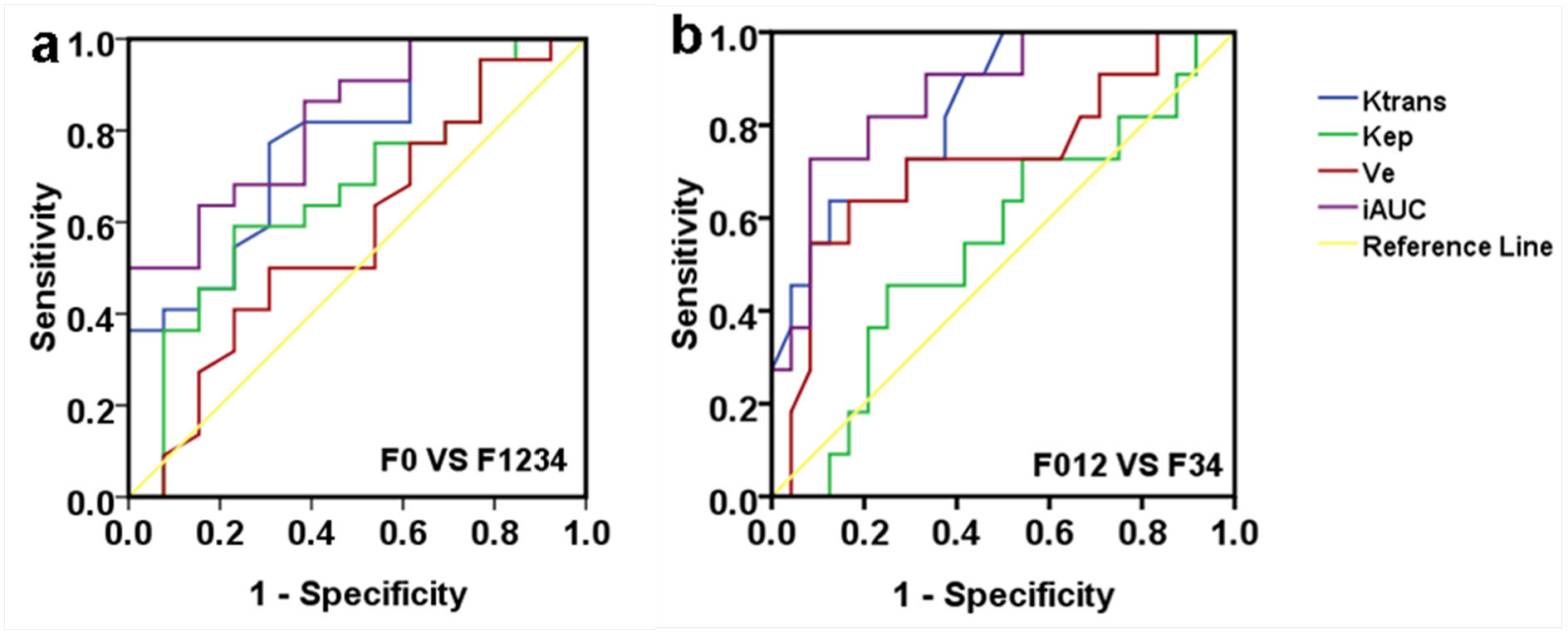
ROC curves for DCE-MRI parameters for diagnosis in rats with (a) fibrosis (stage F1 and greater) and (b) advanced fibrosis (stage F3 and F4).

The results of ROC analysis are shown in Fig 3 and Table 3. K^trans^ and iAUC performed better in the evaluation of liver fibrosis than K_ep_ and V_e_, with an AUROC of 0.773 and 0.882 for fibrosis (stages of F1 and greater), 0.835 and 0.867 for advanced fibrosis (stages of F3 and F4). V_e_ had a moderate utility for diagnosing advanced fibrosis with an AUROC of 0.723.

**Table 3:** Cutoff and performance values of DCE-MRI parameters for diagnosis in rats with any fibrosis and advanced fibrosis.

	F0 vs F1 through F4				F0 through 2 vs F3 and F4			
	K^trans^	K_ep_	V_e_	iAUC	K^trans^	K_ep_	V_e_	iAUC
Cutoff point	0.083	0.300	0.326	7.762	0.098	0.317	0.373	7.816
AUROC	0.773	0.664	0.575	0.882	0.835	0.549	0.723	0.867
Sensitivity	0.773	0.591	0.500	0.500	0.636	0.455	0.636	0.727
Specificity	0.692	0.769	0.692	1.000	0.875	0.750	0.833	0.914
*P* value	0.008	0.109	0.463	0.002	0.002	0.664	0.036	0.001

K^trans^ = transfer constant, K_ep_ = rate constant, V_e_ = extravascular extracellular volume fraction; iAUC = initial area under the gadolinium concentration-time curve; AUROC = area under the receiver operating characteristic curve.

The combination of blood markers (ALT and AST) and DCE-MRI parameters (K^trans^ and iAUC) resulted in improved specificity, but loss of sensitivity for the diagnosis of liver fibrosis (Table 4).

**Table 4:** The results of serial combined examination for blood markers and DCE-MRI parameters.

	F0 vs F1 through F4		F0 through F2 vs F3 and F4	
	Sensitivity	Specificity	Sensitivity	Specificity
K^trans^+ALT	0.527	1.000	0.636	0.969
K^trans^+AST	0.598	0.929	0.636	0.958
iAUC+ALT	0.341	1.000	0.727	0.979
iAUC+AST	0.387	1.000	0.727	0.971

ALT = alanine transaminase; AST = aspartate transaminase; K^trans^ = transfer constant, iAUC = initial area under the gadolinium concentration-time curve.

### Relative Enhancement (RE) Values

**Fig 4:**
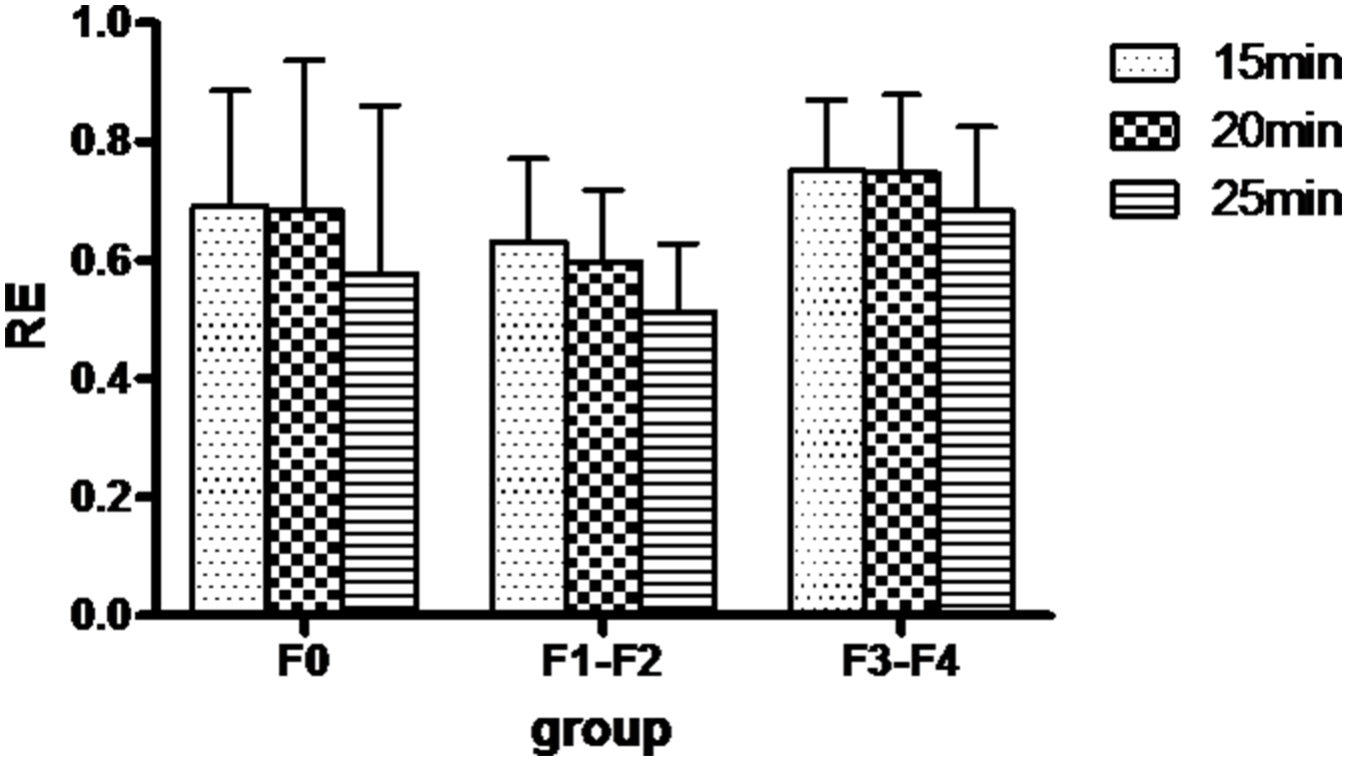
Bar graph of the relative enhancement of different groups at 15, 20, 25 minutes after contrast agent injection.

The RE values for each stage of liver fibrosis at each time point (15, 20, and 25 minutes) are shown in Fig 4. There were no significant differences in RE between the different groups of rats (*P*>0.05).

## Discussion

In this study, we found that K^trans^ and iAUC calculated from DCE-MRI performed well in the detection and staging of CCl_4_-induced liver fibrosis in rats. V_e_ can be used to predict advanced fibrosis. K_ep_ and RE were not able to predict fibrosis. To our knowledge, this is the first study evaluating the utility of K^trans^, K_ep_, V_e_, and iAUC from DCE-MRI with Gd-EOB-DTPA for the detection and grading of liver fibrosis in a rat model.

Activation of hepatic stellate cells is a central event in fibrosis [4]. They produce most of the collagen which deposit in extracellular matrix and leads to formation of the fibrous bands or septa [4, 6]. At the same time, sinusoidal endothelial cells lost the normal fenestrations, transform from the fenestrated hepatic sinusoids into continuous capillaries which is termed capillarization of the sinusoids [25]. Both fibrous bands or septa and sinusoidal capillarization may impede the diffusion of contrast agent [9]. Therefore, it has been postulated that K^trans^ will decrease with the progression of hepatic fibrosis. However, contrary to what has been postulated, we found that K^trans^ increased with fibrosis stage, and the differences in K^trans^ between no fibrosis group and advanced fibrosis group and between mild fibrosis group and advanced fibrosis group were statistically significant. There are several possible reasons for this finding. First, Gd-EOB-DTPA is a low-molecular-weight contrast agent which is less affected by the sinusoidal capillarization than other macromolecular substances, and still can pass from intravascular space to EES quickly in the setting of fibrosis [8, 25]. Second, the speed of blood transfer from sinusoid into EES (reflected by K^trans^) is directly proportional to the pressure in the sinusoid which is increased in fibrosis. One previous study demonstrated an increase arterial flow and arterial fraction in fibrosis [26] which may be the reason for the increasing pressure in sinusoid and accelerated transport. Our results suggest that our current understanding of the pathological mechanism of liver fibrosis is still incomplete, and requires further study.

Secondary to the deposition of collagen and matrix proteins, the volume of EES expands markedly [27]. The EES accounts for approximately 15% of the total fluid space in normal livers, but it can gradually expand to account for more than 50% of the volume in patients with advanced disease [28]. Therefore, V_e_, which is the volume fraction of EES per unit volume tissue [13], should increase with the progression of fibrosis. In our study, V_e_ of advanced fibrosis group was significantly higher than the other two groups, and, V_e_ had a moderate diagnostic accuracy for diagnosing the advanced fibrosis.

IAUC is a semiquantitative measure of the amount of contrast agent delivered to and retained by tissue in a given time period and is considered to be a mixed parameter of both K^trans^ and V_e_ [29]. Many previous studies demonstrated that hepatic mean transit time (MTT) and distribution volume (DV) were significantly increased in advanced fibrosis [25, 30], which would increase the amount of contrast agent delivered to and retained by tissue in a given time period increased. This likely explained the increased iAUC seen with fibrosis in our study.

In our study, we found that with increasing degree of liver fibrosis, the RE decreased initially and then increased, and, RE can not differentiate different stages of fibrosis. However, previous studies showed that RE values by Gd-EOB-DTPA could reflect hepatocyte function and had negative correlation with liver fibrosis stage [23]. A previous study with SD (Sprague Dawley) rats found that the peak signal intensity of liver with Gd-EOB-DTPA were at 5 minutes after administration [31], in our study it was about 2 to 3 minutes after administration. It is possible that sample selection bias, motion artifact, region of interest placement result in the different findings between the two studies.

In previous studies with Gd-EOB-DTPA in clinic, the evaluation of liver fibrosis focus on the slope, the contrast agent uptake rate (*K*_Hep_), liver-to-spleen contrast ratios (LSC) and RE and so on [9, 32–33]. Chen BB [9] found slope and AUC were the best perfusion parameters to predict the severity of liver fibrosis in their study. Norén B [32] found that *K*_Hep_, LSC10, and LSC20 had significant difference between each group. Verloh N [33] found that RE of liver parenchyma is negatively affected by increased severity of liver cirrhosis. However, the quantitative parameters of DCE-MRI were rarely reported to use for Gd-EOB-DTPA study in liver fibrosis.

There were several limitations to our current study. First, we used a modified Tofts model rather than a dual-input two-compartment model, which is considered a better model to investigate the hepatic blood supply. However, the modified Tofts model has been approved by the US food and drug administration (FDA) for investigating liver perfusion state. Second, the sample size was small in this study. The stages of fibrosis were mostly F2 and F3, F1 and F4 stages were relatively few. Third, it is not clear the exact timing of the hepatobiliary phase in rats, which may affect the accuracy of RE measurement. Some studies indicated that enhancement effect of liver parenchyma is the highest at 20 min in healthy controls, and in rat models it is at about 5 to 30 min [22]. One study by Ma et al. selected 20 min in rat models [34]. We choose 15 min, 20 min, and 25 min after administration to scan and calculated RE values in this study.

In conclusion, DCE-MRI with Gd-EOB-DTPA is a promising method for the noninvasive diagnosis and staging of liver fibrosis. Both K^trans^ and iAUC correlate with the stage of liver fibrosis induced by carbon tetrachloride in rats. Future studies using this technique to assess liver fibrosis in patients are warranted.
